# Eco-Innovation in Reusing Food By-Products: Separation of Ovalbumin from Salted Egg White Using Aqueous Two-Phase System of PEG 1000/(NH_4_)_2_SO_4_

**DOI:** 10.3390/polym11020238

**Published:** 2019-02-01

**Authors:** Bin Jiang, Jiaxin Na, Lele Wang, Dongmei Li, Chunhong Liu, Zhibiao Feng

**Affiliations:** Department of Applied Chemistry, Northeast Agricultural University, NO.600 Changjiang Road Xiangfang, Harbin 150030, China; jiangbin@neau.edu.cn (B.J.); 18345342585@163.com (J.N.); 18846127496@163.com (L.W.); lidongmei@neau.edu.cn (D.L.); liuchunhong@neau.edu.cn (C.L.)

**Keywords:** polyethylene glycols, aqueous two-phase system, salted egg white, ovalbumin, liquid chromatography-nano electrospray ionization mass spectrometry

## Abstract

For the purpose of reducing pollution and the rational use of salted egg white, which is a byproduct of the manufacturing process of salted egg yolk, an aqueous two-phase system (ATPS) composed of polyethylene glycols (PEG 1000) and (NH_4_)_2_SO_4_ was investigated to selectively separate ovalbumin (OVA) from salted egg white. With the aim of optimizing the selective separation of OVA using ATPS, a response surface method (RSM) experiment was carried out on the basis of a single-factor experiment. The OVA was characterized by sodium dodecyl sulfate polyacrylamide gel electrophoresis (SDS–PAGE), reversed-phase high-performance liquid chromatography (RP-HPLC), liquid chromatography-nano electrospray ionization mass spectrometry (Nano LC-ESI-MS/MS), and Fourier transform infrared spectroscopy (FT-IR). Under the optimal conditions, the recovery yield of OVA through ATPS (*Y*) and the purity of OVA (*P*) could reach 89.25% and 96.28%, respectively. In conclusion, OVA was successfully separated from the salted egg white by PEG/(NH_4_)_2_SO_4_ ATPS.

## 1. Introduction

Increased global industrialization and environmental pollution have brought about irreversible environmental damage. The international community has been working to establish a circular economy to minimize resource use and waste. For this purpose, eco-innovation has been introduced. Eco-innovation focuses on the demand for sustainable development. It proposed translating waste and byproducts into resources through shifts in technology [1]. It has been recognized that the waste and byproducts in the food industry can be used as an abundant and cheap source of functional substances. Carbone et al. used white grape pomace aqueous extract as both a reducing and a capping agent to synthetize dendritic silver nanostructures, composed of silver nanoparticles [2]. Jiang et al. separated the antioxidant peptides from whey protein isolate (WPI) hydrolysate, which is a by-product of the manufacturing process of casein [3]. Cacciotti et al. reported that multifunctional eco-sustainable systems, based on poly (lactic acid) as the biopolymeric matrix, could be produced using spent coffee grounds extract as an oxygen scavenger and diatomaceous earth as a reinforcing filler in response to an urgent market demand for green and eco-sustainable products [4]. Effective utilization of by-products in production processes not only save resources but also reduces environmental pollution. Therefore, it is necessary and valuable to explore new technologies and new ideas to reprocess and utilize the waste and byproducts in the production of new commercially valuable products.

Ovalbumin (OVA), a typical globular protein, accounts for the major content of egg white protein at approximately 54% [5]. It consists of 385 amino acids (45 kDa) [6] with an isoelectric point (pI) of about 4.5. In addition to it being an ideal protein source with a well-balanced amino acid composition, OVA displays several functional properties, including emulsification, heat-setting, and foaming. Furthermore, OVA has been reported as an effective drug carrier [7] and it can be applied for tumor suppression owing to its tumor necrosis releasing factors [8]. 

OVA has been extracted and purified from egg white proteins by several methods, including salt precipitation, various chromatography methods, electrophoresis, and ultrafiltration. It was first isolated from egg white in the 1900s by ammonium sulfate precipitation [9]. The method based on the experiment was further researched by Warner and Weber et al. [10]; however, the low purity and irreversible unfolding caused by the high salt concentration implied that ammonium sulfate precipitation was not a satisfactory method for extracting OVA. A chromatographic method was first used to extracted OVA from egg white in 1960 [11]. Since then, some chromatography methods, such as anion exchange chromatography [12], reversed-phase high-performance liquid chromatography, and gel-permeation chromatography [13], were developed to extract OVA. Nevertheless, it is difficult for chromatographic methods to be used in the industry due to their low yield, expensive operating costs, and high equipment requirement. OVA was separated by electrophoresis from egg white [14]; however, denaturation was observed during the extracting process. Ultrafiltration was applied to separate OVA [15]; however, ultrafiltration membranes lack selectivity and are easily contaminated during ultrafiltration [16]. Therefore, it is necessary to develop an economical and simple method to isolate OVA from egg white.

An aqueous two-phase system (ATPS) has been widely studied in the past decades due to its advantages, such as enhanced selectivity, process integration, scale-up, low toxicity, continuous operation, and biocompatibility, and it was effectively applied for the extraction and purification of some biomolecules [17]. The most common ATPSs are formed by polymers (usually polyethylene glycol (PEG)) and a salt (e.g., phosphate, citrate or sulfate) [17]. Hence, ATPSs composed of PEG and salt were applied to extract proteins from egg white. In 2006, the separation of lysozyme from egg white by an ATPS composed of polyethylene glycol (PEG 6000) and sodium sulfate was reported by Su et al. [18]. In 2009, Fabíola et al. [19] discovered that ovomucoid could be extracted from egg white by an ATPS consisting of PEG 1500 and sodium carbonate, and the extraction of avidin was reported by ATPS in 2013 [20]. In 2016, Pereira et al. [21] reported that an ATPS consisting of PEG 400 and potassium citrate/citric acid (pH 7.0) was developed to extract OVA from egg white, with a recovery yield of 65%. However, it was found that the system could not extract OVA from salted egg white by our preliminary experiments.

Salted eggs are a famous traditional egg product in China. Salted egg yolk is widely used in a variety of food products, such as mooncakes [22]. Salted egg white is a byproduct of the manufacture process of salted egg yolk, and is normally treated as a waste. The annual output of salted egg white exceeds 10,000 tons. Salted egg white thus has a tremendous annual output without a reasonable application. The direct discharge of salted egg white containing a large amount of salt and protein not only wastes protein resources, but also pollutes the environment. At present, the main treatment methods are its direct use as a food additive, desalting treatment, enzymatic treatment, and the extraction of lysozymes [23]. Based on the concept of eco-innovation, we tried to separate OVA from salted egg whites. The aim of this study was to develop a simple, inexpensive, and efficient process for the separation of OVA from salted egg white, so as to improve the utilization of protein resources and reduce pollution. With this goal, the separation of OVA from salted egg white by PEG 1000/ammonium sulphate ATPS was studied for the first time, and sodium dodecyl sulfate polyacrylamide gel electrophoresis (SDS–PAGE), reversed-phase high-performance liquid chromatography (RP-HPLC), liquid chromatography-nano electrospray ionization mass spectrometry (Nano LC-ESI-MS/MS), and Fourier transform infrared spectroscopy (FT-IR) were applied to characterize the obtained OVA.

## 2. Materials and Methods

### 2.1. Instruments

Chromatographic analysis was carried out on a Waters e2695 high performance liquid chromatograph (Waters, Waters Corporation, Milford s, Massachusetts, USA) equipped with C_8_ and C_18_ columns and an ultraviolet visible detector (Waters, Waters Corporation, Milford, Massachusetts, USA). An SC-3610 low-speed centrifuge was utilized for auxiliary phase separation (Anhui Zhongke Zhongjia Scientific Instrument Co., Ltd., Hefei, China). The employed ultraviolet-visible spectrophotometer was from Beijing Purkinje General Instrument Co., Ltd (Beijing, China). The employed A150011 vortex mixer was from Nanjing Jiajun Biological Co., Ltd (Nanjing, China). An FE201EL20 pH meter was utilized to determine the pH of the solutions, and an AL-04 electronic analytical balance (Mettler Toledo Instruments Co., Ltd., Shanghai, China) was used for weighing.

### 2.2. Reagents

Polyethylene glycol (PEG), with an average molecular weight (MW) of 1000 Da, was from Sinopharm Chemical Reagent Co., Ltd. (Shanghai, China). The SDS–PAGE electrophoresis kit was from Beijing Solarbio Technology Co., Ltd. (Beijing, China). Trifluoroacetic acid (TFA) and acetonitrile were chromatographically pure (Dikma, Beijing, China). OVA from hen egg white was obtained from SigmaUSA. Salted eggs were purchased in a supermarket. Other reagents used were of analytical grade.

### 2.3. Preparation of ATPS and Purification

The ATPSs were prepared by mixing the appropriate amount of PEG 1000 in a stock solution of ammonium sulfate in 10 mL graduated centrifuge tubes. Next, 10% (*v*:*v*) salted egg white solution was added to each system. The pH of the mixture was adjusted using NaOH (10 mol/L). The systems were vigorously mixed by a vortex mixer and centrifuged at 716 × *g* for 10 min to induce phase separation. The phase volumes were measured and then the top phases were collected for further analysis. 

Salted egg white solution and top phases of ATPS were dialyzed (13,000 Da) overnight against deionized water to remove the salt and PEG 1000. They were then lyophilized to obtain the purified protein.

### 2.4. Definition of the Distribution of Protein in ATPS

The concentrations of protein in top phases of the ATPSs and salted egg white solution were determined by the Bradford method [24]. Using BSA (albumin from bovine serum) as a standard, the samples were measured at 595 nm. The separation parameters were calculated using the following formulae:

(1) $$Y=\frac{M_{\mathrm{OVA}}}{M}\times 100\%$$

(2) $$P=\frac{M_{\mathrm{OVA}}}{M_{\mathrm{TotalProteins}}}\times 100\%$$

where *Y* is the recovery yield of OVA in ATPS, *P* is the purity of OVA, *M*_OVA_ and *M* are the weight measured by RP-HPLC of OVA in the top phase and salted egg white solution, respectively. *M*_Total Proteins_ was the total protein in the top phase.

### 2.5. Experimental Design

In order to optimize the interaction between the separation parameters and parallel factors, a response surface method (RSM) experiment was carried out on the basis of a single-factor experiment. The effects of various factors, including *X*_1_ (the mass fraction of PEG), *X*_2_ (the mass fraction of (NH_4_)_2_SO_4_), *X*_3_ (the pH value), and *X*_4_ (the salted egg white solution), were studied. The range of coded variable levels was determined by a single factor experiment. The experimental design is shown in Table 1. *Y* and the *P* were taken as the responses of the experiments. Each experiment was replicated three times.

A second-order polynomial equation model was used to fit the *Y* and *P* data with interaction terms, as given below:

*M* = *A*_0_ + *A*_i_Ʃxi + *A*_ij_Ʃxixj + *A*_ii_Ʃxi2 (i ≠ j) (3)

where *M* is the predicted response value; *A*_0_, *A*_i_, *A*_ij_, and *A*_ii_ are the regression coefficients of the model for the intercept, linear, cross-product, and quadratic terms, respectively; and *x*i is the variable under study [25].

### 2.6. Electrophoresis

Twelve percent bis-acrylamide homogeneous gel was selected as the SDS-PAGE electrophoresis condition to identify proteins. The gel was run at a constant voltage of 80 V for about 20 min for stacking the gel, and at 120 V for 55 min for separating the gel. After the end of the electrophoresis, the tape was sequentially stained with Coomassie Brilliant Blue R-250 for 30 min and decolorized with eluent [26].

### 2.7. RP-HPLC

RP-HPLC was used to analyze the protein in the top phase before and after purification. The conditions for RP-HPLC were as follows: Waters C_8_ column (4.6 mm × 150 mm), 0.05% TFA in acetonitrile as mobile phase A, 0.05% aqueous TFA as mobile phase B, flow rate of 1.00 mL/min, and detection wavelength of 280 nm. Gradient elution was programmed as follows: the concentration of mobile phase A was increased from 7% to 70% in 17 min and decreased to 7% before 22 min [27].

### 2.8. FT-IR

A 2 mg sample of powder was mixed with 200 mg of potassium bromide powder, which was ground under an infrared baking lamp for 10 min and pressed in a mold. The measurement parameters were: resolution 4 cm^−1^, wave number accuracy 0.01 cm^−1^, 64 scanning times, ambient temperature 25 °C, infrared scanning wavenumber 400 cm^−1^–4000 cm^−1^. The sample was detected by FT-IR. The experiment was repeated three times [28]. After obtaining the infrared spectra, the protein conformation was analyzed by Peak Fit v4.12 software.2.9. *Nano LC-ESI-MS/MS*.

Nano LC-ESI-MS/MS analysis of a digested protein sample was carried out by an HPLC system with a C_18_ column (75 μm × 80 mm). The HPLC solvent A was 97.5% water, 2% acetonitrile, and 0.5% formic acid. The HPLC solvent B was 9.5% water, 90% acetonitrile, and 0.5% formic acid. The gradient elution process is described as follows. The concentration of mobile phase B was improved from 2% to 90% within 60 min. The column flow rate was around 800 nanoliter per minute after splitting. The typical sample injection volume was 3 µL. The HPLC system was on-line coupled with a linear ion trap mass spectrometer (LTQ, Thermo) so that a sample eluted from the HPLC column was directly ionized by an electrospray ionization (ESI) process and entered into the mass spectrometer. The ionization voltage was often optimized in the instrument tuning process and normally in a range of 1.5–1.8 kv. The capillary temperature was set at 100 °C. The mass spectrometer was set at the data-dependent mode to acquire MS/MS data via a low energy collision induced dissociation (CID) process. The default collision energy was 33% and the default charge state was 3. One full scan with one microscan with a mass range of 350 amu to 1650 amu was acquired, followed by nine MS/MS scans of the nine most intense ions with a full mass range and three microscans. The dynamic exclusion feature was set as follows: repeat count of 1 and exclusion duration of 1 min. The exclusion width was 4 Da.

## 3. Results and Discussion

### 3.1. Single-Factor Variable Analysis

**Figure 1:**
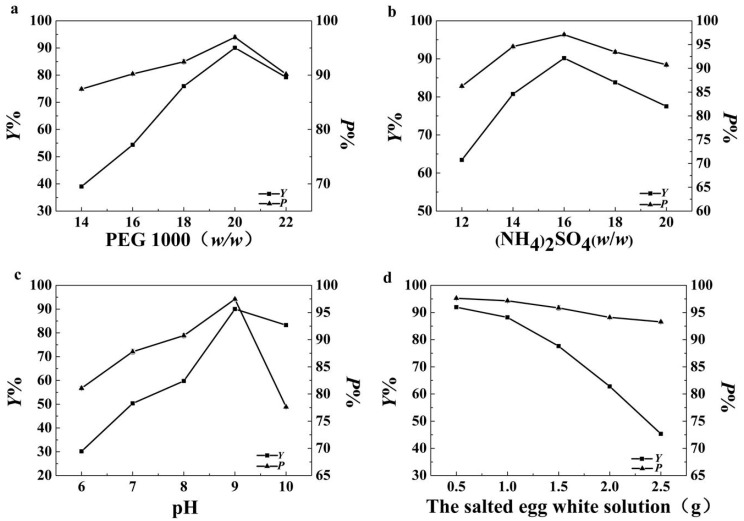
Comparison of single factor results. (**a**) Effect of the concentration of PEG 1000 on the extraction;(**b**) effect of the concentration of (NH_4_)_2_SO_4_on the extraction; (**c**) effect of pH on the extraction; (**d**) effect of the weight of the salted egg white solution on the extraction.

The effect of the mass fraction of PEG 1000 and (NH_4_)_2_SO_4_, the pH and the weight of the salted egg white solution were chosen for study. Considering the efficiency of the process, 0.5 g salted egg white was selected as the minimum amount of raw material added. Based on Y and P, the optimal conditions of the ATPS were chosen for further investigation. The results are indicated in Figure 1.

The effect of the mass fraction of PEG 1000 is described in Figure 1a. The trend of Y and P changed in a similar way. Both Y and P reached the maximum value when the mass fraction of PEG was 20% (*w*/*w*), which meant that almost all of the OVA was separated into the top phase, while other proteins in the egg white were left in the bottom phase. The difference in protein distribution between the top and bottom phases was perhaps due to the properties of the protein, such as shape, molecular weight, volume, and surface area [29]. Electrostatic interactions, hydrophobic interaction, and the salting-out effect were the main driving forces of the protein distribution in the ATPS [17]. When the mass fraction of PEG ranged from 14% to 20%, the change of the concentration of PEG affected the hydrophobicity of the top phase. The more hydrophobic OVA probably interacted preferentially with the more-hydrophobic PEG-rich phase, enhancing the extraction of OVA to the top phase [30]. At mass fractions above 20%, the protein affinity to the polymer-rich phase decreased, mainly due to an increase in the steric exclusion of proteins from that phase. Therefore, protein would tend to partition to the salt-rich phase [31]. Another reason for this phenomenon was that a high PEG concentration increased the excluded volume of PEG, which in turn reduced the solvent volume fraction. Thus, protein was excluded by the phase-forming polymer [25].

The effect of the mass fraction of (NH_4_)_2_SO_4_ is described in Figure 1b. Y and P increased with the increase of the concentration of (NH_4_)_2_SO_4_ until the mass fraction reached 16%. With the increase of the (NH_4_)_2_SO_4_ concentration, the ionic strength and the hydrophobicity of the salt-rich bottom phase increased [32]. The phase hydrophobicity effect and the salting out effect played important roles in the partitioning of protein. The salting-out effect moveds the hydrophobic OVA from the salt-rich phase to the polymer-rich phase [33]. At mass fraction above 16%, Y and P in the top phase decreased. One possible reason for this was that the protein precipitated at the interphase was discarded [22]. 

The effect of the pH is described in Figure 1c. When the pH was raised from 6 to 9, Y and P increased. The distribution of proteins in the ATPS was affected by pH, which could be attributed to the surface properties and charge of the protein being altered. When the pH is higher than the isoelectric point (pI), protein carries a negative charge; otherwise, it carries a positive charge. The net charge of the protein at the isoelectric point is zero [33]. When pH is higher than 4.5, OVA carries a negative charge. Negatively charged biomolecules prefer the organic phase in higher pH systems. Higher pH values than the pI of protein induce an affinity towards the PEG-rich phase because of the positive dipole moment [34]. At pH levels above 9, P decreased, meaning that protein with a higher impurity entered the top phase. At pH 9, both Y and P reached the maximum value and OVA retained biological activity [35]. Thus, the optimal values for the partition parameters were obtained at pH 9.

The effect of the weight of salted egg white solution is described in Figure 1d. As the weight of the salted egg white solution increased, Y decreased sharply and P changed slowly, which meant that the increase in the weight of the protein solution hardly affected the selectivity of OVA of the top phase and seriously affected the recovery yield of OVA. Moreover, precipitate was observed at the interface between the two phases when the weight of the protein solution reached 1.5 g. The PEG-rich phase was more hydrophobic than the salt-rich phase. 

Hence, little free water was available in the PEG-rich phase for interactions. That induced the precipitation of hydrophobic protein at the interface between the two phases [36]. Thus, excess salted egg white solution could result in a significant decrease of Y. With the aim of improving processing efficiency, 1.0 g salted egg white solution was selected as the optimal condition.

From the above analysis, it could be concluded that the best single-factor conditions were as follows: PEG 20% (*w*/*w*), ammonium sulfate 16% (*w*/*w*), pH 9, and 1.0 g salted egg white solution.

### 3.2. Response Surface Analysis

#### 3.2.1. Statistical Analysis and Model Fitting

Response surface methodology (RSM) was used for the final optimization of significant factors [16]. This methodology is useful in the determination of optimal operating conditions and significant independent factors or their interactions with dependent output responses in a multivariate complex system (e.g., ATPS) [25,37,38]. Single factor experiments can roughly find the range of optimal conditions, and RSM can determine the optimal conditions. To better verify the results of the single-factor experiments, RSM is an effective mathematical statistical method.

The experimental results of the response surface design are shown in Table 2. Regression analysis was performed on the experimental data obtained using Design-Expert 8.0.6 software, and the equations for predicting the *Y* and *P* values of OVA were obtained, which were given as follows:

*Y*(%) = 88.96 + 1.98 × *A* − 0.31 × *B* + 7.54 × *C* − 1.84 × *D* − 4.00 × *A* × *B* − 1.57 × *A* × *C* + 0.34 × *A* × *D* − 0.91 × *B* × *C* + 3.69 × *B* × *D* + 0.36 × *C* × *D* − 10.70 × *A*^2^ − 15.49 × *B*^2^ − 11.27 × *C*^2^ − 12.25 × *D*^2^ (4)

*P*(%) = 95.81 − 1.13 × *A* − 0.017 × *B* − 2.29 × *C* + 0.21 × *D* + 0.20 × *A* × *B* − 0.30 × *A* × *C* + 0.57 × *A* × *D* − 1.17 × *B* × *C* − 1.00 × *B* × *D* − 0.78 × *C* × *D* − 5.28 × *A*^2^ − 7.67 × *B*^2^ − 10.24 × *C*^2^ − 1.87 × *D*^2^ (5)

#### 3.2.2. Analysis of Variance

Analysis of variance (ANOVA) for the Y and P models is shown in Table 3 and Table 4, respectively. The regression models were highly significant (*p*_1_ < 0.01, *p*_2_ < 0.01), while the lack-of-fit tests were not significant (*p*_1_ = 0.0725 > 0.05, *p*_2_ = 0.3296 > 0.05). The determination coefficients (*R*_1_^2^ and *R*_2_^2^) of the predicted models were 0.9918 and 0.9558, indicating a high degree of correlation between the true and predicted values. Thus, the models explained the response adequately and could be used to analyze and predict the optimal separation conditions of OVA.

#### 3.2.3. Interactive Analysis

**Figure 2:**
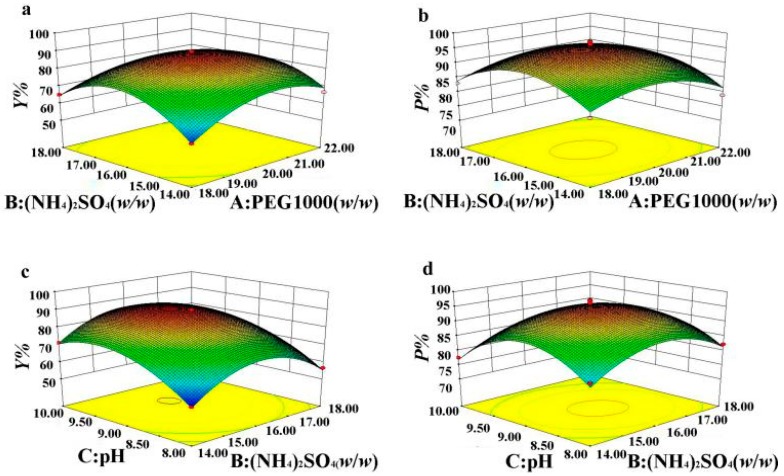
Response surface plots for Y (**a**,**c**) and P (**b**,**d**) of OVA.

The relationship between response and experimental levels of each variable was visualized in a three-dimensional (3D) response surface plot, which provided a method to directly observe the interactions between the two test variables [39]. The factors influencing the Y and P values of OVA are shown as the response surface plots in Figure 2. 

Figure 2a,b show that at a specific level of PEG in the ATPS, Y and P first increased and then decreased with the increase of the mass fraction of (NH_4_)_2_SO_4_. Similarly, at a specific level of (NH_4_)_2_SO_4_ in the ATPS, Y and P first increased and then declined with the increase of the mass fraction of PEG. When PEG and (NH_4_)_2_SO_4_ reached specific levels, the maximum values of Y and P were obtained. It could be inferred that a weak ionic strength and a weak hydrophobic interaction were obtained in the ATPS when both the PEG value and the (NH_4_)_2_SO_4_ value were at low levels, which was not conducive to the movement of protein to the top phase. With the increase of PEG and (NH_4_)_2_SO_4_, the ionic strength or hydrophobic interaction gradually increased, and hydrophobic OVA was more easily distributed into the top phase and impurity proteins were retained in the bottom phase, causing Y and P to increase. However, as the PEG value and the (NH_4_)_2_SO_4_ value increased, the volume of the top phase decreased and the ionic strength in the bottom phase increased, causing the protein to precipitate at the interface of the two phases [22]. 

Figure 2c,d, showed that the trends of Y and P are similar to those in Figure 2a,b; the lower or higher pH values and the (NH_4_)_2_SO_4_ value were not conducive to the movement of OVA to the top phase. With the increase of the pH and (NH_4_)_2_SO_4_ values, the ionic strength gradually increased, causing more hydrophobic parts on the protein surface to be exposed. Thus, more protein was extracted to the hydrophobic phase. However, excessively high ionic strength and the salting-out effect not only reduced Y, but also decreased the selectivity of the top phase for OVA, resulting in a reduction of P.

#### 3.2.4. Validation of the Best Extraction Conditions

According to the results of Box-Behnken (BBD), the optimal conditions were obtained when the mass fraction of PEG 1000 and (NH_4_)_2_SO_4_, the pH of the system and the weight of the salted egg white solution were 19.99%, 15.98%, 9.06, and 0.97 g, respectively. Under these conditions, the Y and P value of OVA could reach 89.46% and 95.61%, respectively. In order to facilitate the operation, the predicted optimal process conditions were amended as follows: mass fraction of PEG 1000 20%, mass fraction of (NH_4_)_2_SO_4_ 16%, pH 9, and salted egg white solution 1.0 g. Under these conditions, Y was 89.25 ± 0.76% and P was 96.28 ± 0.97%. There was no significant difference between the result and the predicted value, indicating that the model was reliable. 

### 3.3. Characterization of Ovalbumin Extracted Directly from Salt Egg White

**Figure 3:**
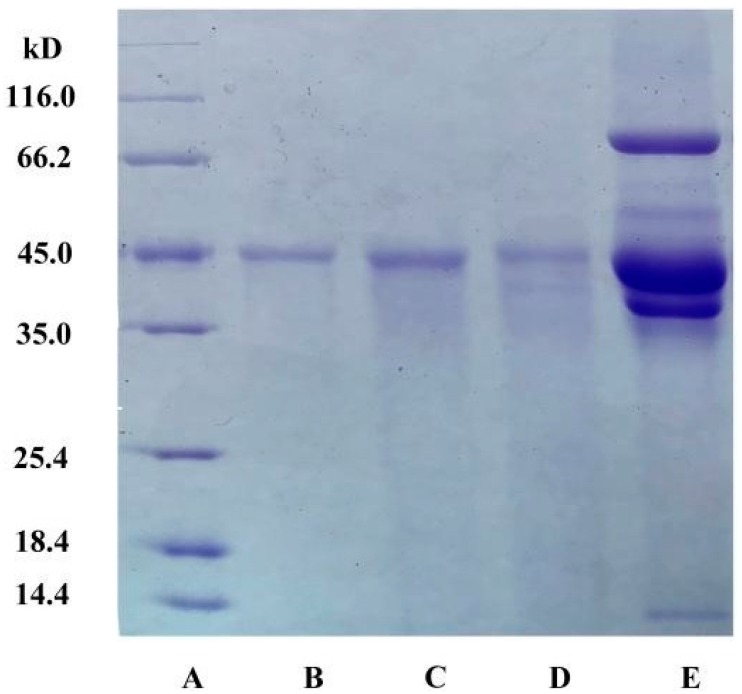
SDS–PAGE of OVA. (**A**) molecular mass standards; (**B**) sample (10 μg) extracted by ATPS; (**C**) sample (20 μg) extracted by ATPS; (**D**) OVA standard (10 μg); (**E**) the salted egg white solution (10 μg).

Figure 3 shows the SDS-PAGE of OVA (from the salted chicken egg white after the ATPS process) found in the top phase (lanes B, C), OVA standard (lane D), and the salted egg white solution (lane E).

The electrophoretic analysis of the salted egg white solution showed multiple bands (lane E), while the SDS–PAGE of the OVA after the ATPS extraction showed one band (lanes B, C) with a major band at 45 kDa, meaning that only one major protein was extracted to the top phase. The major band was similar to the ovalbumin standard (lanes D). Thus, it was inferred that the major band could be OVA.

**Figure 4:**
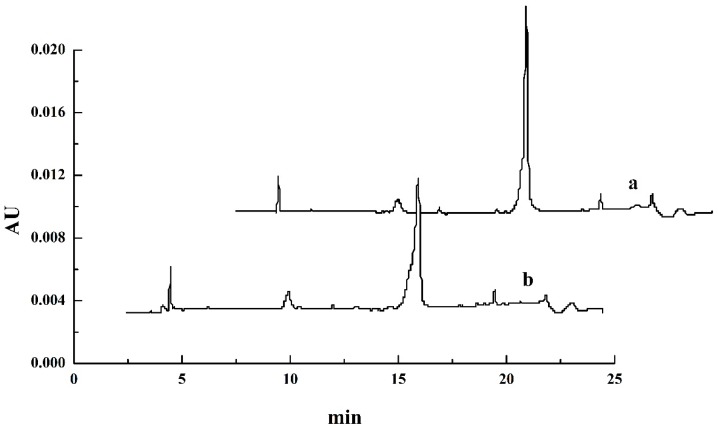
RP-HPLC chromatograms of the OVA standard (**a**) and the top phase of the ATPS (**b**).

However, the sensitivity of electrophoresis was limited, and it was difficult to detect proteins with a similar molecular weight. Therefore, the protein in the top phase could not be accurately defined as OVA. Chromatography was further applied to characterize OVA. Under the optimized conditions described above, the top phase of the ATPS and the OVA standard were measured by RP-HPLC. The obtained chromatograms are shown in Figure 4. The retention times of the peaks in the chromatogram of the top phase of the ATPS were all approximately 2 min and 13.5 min; these retention times correspond to the position of the OVA standard. Thus, it could be concluded that only OVA was presented at the top phase since no other peaks were identified, meaning that pure OVA was obtained by the ATPS. These RP-HPLC chromatograms also confirmed that there was no significant OVA aggregation or fragmentation under the studied conditions, since no other peaks were identified [19]. OVA from the top phase was further determined by NanoLC-ESI-MS/MS.

Nano LC-ESI-MS/MS is the most sensitive and reliable method for identifying gel-separated protein tapes. Moreover, multiple protein components in a single protein tape can be identified using this method.

Table 5 and Table 6 show the results of the OVA sample and the peptide of the OVA determined by Nano LC-ESI-MS/MS. Four proteins were detected, including OVA, alpha-1-acid glycoprotein, DNA topoisomerase 2-beta, and ovomucoid. The relative abundance of OVA was 98.4%, meaning that OVA was the main protein in the top phase of the ATPS.

**Figure 5:**
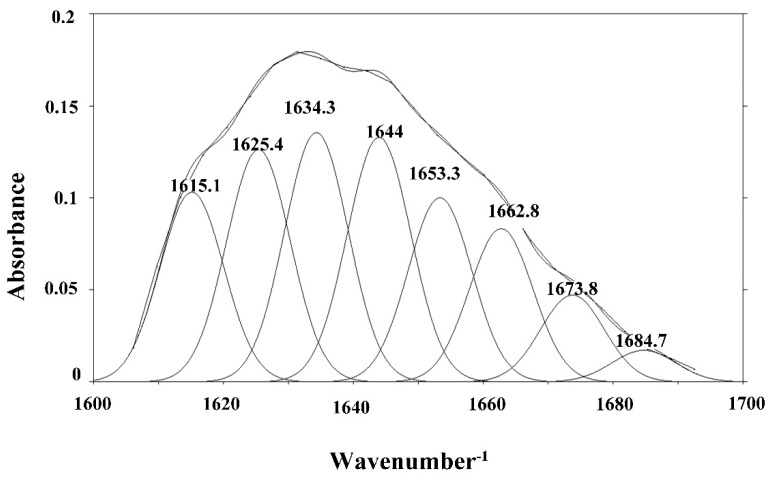
Fitting FTIR chart for the OVA standard.

**Figure 6:**
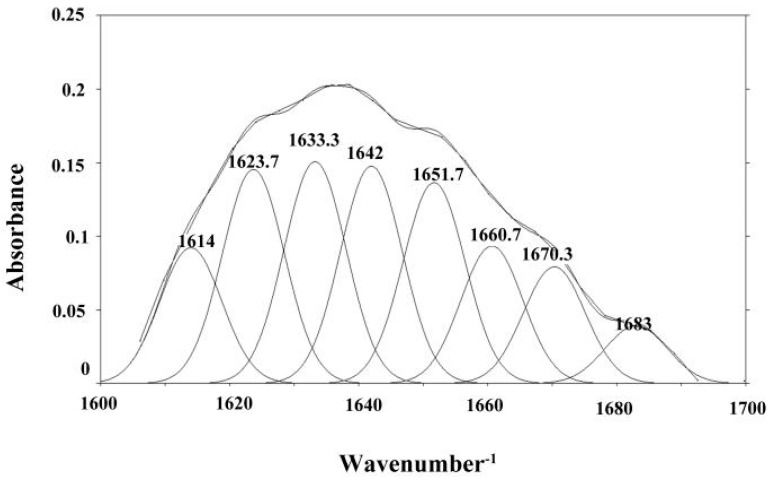
Fitting FTIR chart for OVA from the top phase of the ATPS.

**Figure 7:**
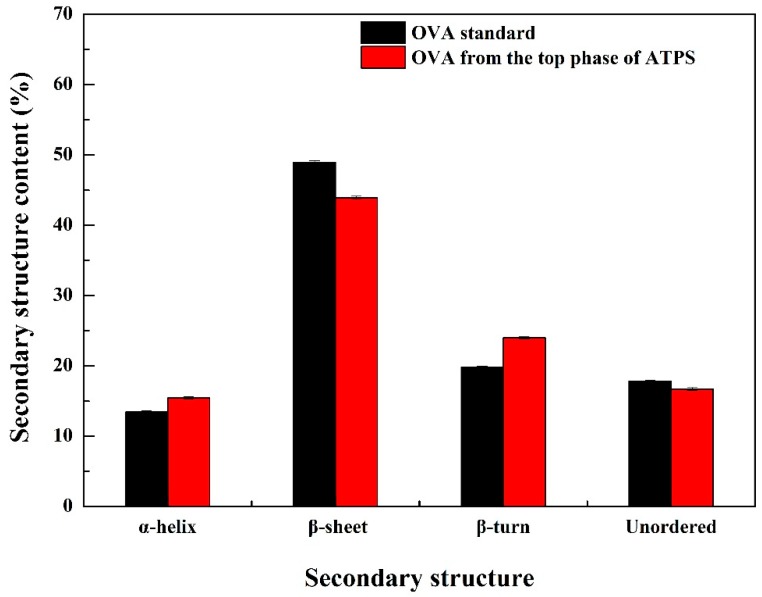
The content of the secondary structure for OVA obtained by different sources.

The structure of the protein in the top phase of ATPS was further characterized by FT-IR. Secondary structure information of proteins was analyzed by FT-IR and quantitatively analyzed based on the energy absorption bands of their chemical bonds or functional groups. Most analyses were between 1690 and 1600 cm^−1^ for the specific investigation of the protein structure which corresponded to the vibration of the amide I group. At present, the combination of Deconvolve Gaussian IRF and second derivative is commonly used to analyze the amide I absorption band [40,41]. Referring to the literature, the corresponding relationship between each sub-peak and secondary structure was as follows: α-helix (1650~1660 cm^−1^); β-sheet (1610~1640 cm^−1^); *β*-turn (1660~1700 cm^−1^); unordered (1640~1650 cm^−1^) [42,43]. PeakFit v4.12 software was used to analyze the original infrared map amide I absorption band of the sample and OVA standards, and the fitted map was obtained after baseline correction, Deconvolve Gaussian Instrument Response Functions (IRF) deconvolution, and second derivative fitting. The results are shown in Figure 5 and Figure 6. According to the corresponding relationship between each sub-peak and the secondary structure, the integral area was calculated, and the relative percentage of the secondary structure was obtained, as shown in Figure 7. The results showed that α-helix, β-sheet, β-turn, and unordered were not obviously changed, which indicated that there was no change in the spatial structure of the protein in the ATPS separation process.

## 4. Conclusions

In this study, the single-step extraction of OVA from salted egg white using an ATPS containing PEG 1000/(NH_4_)_2_SO_4_ was investigated successfully. The optimum ATPS conditions were obtained using response surface methodology and a Box–Behnken experimental design. Under optimum conditions, including 20% PEG 1000 (*w*/*w*), 16% (NH_4_)_2_SO_4_ (*w*/*w*), pH 9, and 1 g salted egg white solution, maximum Y and the P values of 89.25 ± 0.76% and 96.28 ± 0.97%, respectively, could be reached. Separating ovalbumin through the ATPS proved to be efficient based on the determination of SDS–PAGE, RP-HPLC, Nano LC-ESI-MS/MS, and FT-IR analyses. Therefore, it was suggested that the ATPS could be a valuable protocol for the separation of OVA from salted egg white. It might be an effective and sustainable way to reduce environmental pollution and improve the utilization of salted egg white. The goal of extracting OVA from salted egg white was achieved to some degree; however, purification method need to be further studied.

## Figures and Tables

**Table 1:** Factors and levels in the response surface design used for the optimization of ovalbumin (OVA) extraction by an aqueous two-phase system (ATPS).

Variables	Coded Variable Levels		
	−1	0	1
*X*_1_ PEG1000 (*w*/*w*)%	18	20	22
*X*_2_(NH_4_)_2_SO_4_ (*w*/*w*)%	14	16	18
*X*_3_ pH	8	9	10
*X*_4_ Salt egg white solution/g	0.5	1	1.5

**Table 2:** Box-Behnken (BBD) and the results (means of triplicate tests) for the recovery yield and purification factor of OVA.

Number	*X*_1_ PEG 1000 (*w*/*w*) %	*X*_2_ (NH_4_)_2_SO_4_ (*w*/*w*) %	*X*_3_ pH	*X*_4_ Egg White Solution (g)	Protein Recovery (%)	Purity (%)
1	−1	−1	0	0	57.17	82.3
2	−1	1	0	0	65.09	83.05
3	1	−1	0	0	66.66	78.88
4	−1	0	1	0	73.47	80.35
5	0	−1	−1	0	54.59	80.23
6	1	0	1	0	74.82	78.14
7	0	1	−1	0	56.44	82.25
8	0	0	0	0	88.92	95.01
9	0	0	0	0	89.34	93.56
10	0	1	0	1	62.54	86
11	0	−1	1	0	71.27	77.59
12	0	−1	0	−1	67.58	86.23
13	0	1	0	−1	58.42	87.32
14	−1	0	−1	0	56.35	83.53
15	0	0	0	0	88.68	97.01
16	1	1	0	0	58.56	80.42
17	0	0	1	−1	74.1	80.23
18	0	0	0	0	89.94	96.21
19	0	0	0	0	87.92	97.25
20	1	0	0	−1	71.55	87.65
21	0	−1	0	1	56.95	88.93
22	0	1	1	0	69.48	74.92
23	0	0	1	1	71.38	78.83
24	0	0	−1	−1	58.41	83.62
25	0	0	−1	1	54.26	85.33
26	−1	0	0	−1	66.32	90.95
27	1	0	0	1	67.86	89.23
28	1	0	−1	0	63.96	82.53
29	−1	0	0	1	61.27	90.24

**Table 3:** The variance analysis of the fitted quadratic polynomial prediction model of Y.

Source	Sum of Squares	df	Mean Square	*F*	*p*_1_-Value
Model	3570	14	255.04	121.60	<0.0001
Residual	29.36	14	2.10		
Lack of fit	27.10	10	2.71	4.78	0.0725
Pure error	2.27	4	0.57		
Cor total	3599.95	28			
CV%			2.12		
*R*_1_^2^			0.9918		

**Table 4:** The variance analysis of the fitted quadratic polynomial prediction model of P.

Source	Sum of Squares	df	Mean Square	*F*	*p*_2_-Value
Model	1046.69	14	74.76	21.64	<0.0001
Residual	48.36	14	3.45		
Lack of fit	38.99	10	3.90	1.66	0.3296
Pure error	9.38	4	2.34		
Cor total	1095.09	28			
CV%			2.18		
*R*_2_^2^			0.9558		

**Table 5:** The test results of the OVA sample by Nano LC-ESI-MS/MS.

Hits	Protein Mass	No. of Peptide	Protein	UniprotKB Databases	Relative Abundance	Probability	No. of Unique Peptide
1	43195.66	174	OVA of chick	P01012	98.4%	99.0%	17
2	22535.07	7	Alpha-1-acid glycoprotein of chick	Q8JIG5	1.5%	99.0%	4
3	184156.91	1	DNA topoisomerase 2-beta of chick	O42131|	0.0%	85.9%	1
4	3659.67	1	Ovomucoid of chick	P01005	0.0%	82.1%	1

**Table 6:** The peptide of the OVA sample determined by Nano LC-ESI-MS/MS.

Scan No.	Peptide Mass	Peptide Sequence of Protein from Chick	Peptide Probability
6370	956.58	TQINKVVR	89.5%
6646	1772.89	ISQAVHAAHAEINEAGR	92.6%
6905	887.56	IKVYLPR	78.1%
6967	1554.71	AFKDEDTQAMPFR	95.2%
7040	1580.71	LTEWTSSNVMEER	93.2%
7051	943.53	DILNQITK	91.3%
7156	1686.83	GGLEPINFQTAADQAR	94.9%
7238	2007.94	EVVGSAEAGVDAASVSEEFR	95.7%
7277	1246.62	ADHPFLFCIK	83.1%
7285	1344.73	HIATNAVLFFGR	95.7%
7486	1521.79	YPILPEYLQCVK	90.7%
7626	2280.17	DILNQITKPNDVYSFSLASR	93.4%
7636	1481.75	PVQMMYQIGLFR	93.5%
7746	2283.14	VTEQESKPVQMMYQIGLFR	87.9%
9056	2459.31	NVLQPSSVDSQTAMVLVNAIVFK	92.9%
9062	1857.96	ELINSWVESQTNGIIR	94.5%
9081	3032.51	VHHANENIFYCPIAIMSALAMVYLGAK	85.1%
